# Supplementary material for: Simultaneous Probing of Metabolism and Oxygenation of Tumors In Vivo Using FLIM of NAD(P)H and PLIM of a New Polymeric Ir(III) Oxygen Sensor
Source: Int J Mol Sci. 2022 Sep 6;23(18):10263. doi: 10.3390/ijms231810263 (PMC9499414; doi:10.3390/ijms231810263)
Supplement: Supplementary file 1 [file ijms-23-10263-s001.zip › ijms-1881874-supp..pdf]

Supplementary Material for the article

Simultaneous probing of metabolism and oxygenation of tumors in vivo using FLIM of NAD(P)H and PLIM of a new polymeric Ir(III) oxygen sensor

Yulia P. Parshina [1,†], Anastasia D. Komarova [2,3†], Leonid N. Bochkarev [1], Tatyana A. Kovylina [1], Anton A. Plekhanov [2], Larisa G. Klapshina [1], Aleksey N. Konev [1], Artem M. Mozherov [2], Ilya D. Shchechkin [2,3],
Marina A. Sirotkina [2], Vladislav I. Shcheslavskiy [2,4] and Marina V. Shirmanova [2,*]

[1] G. A. Razuvaev Institute of Organometallic Chemistry, Russian Academy of Sciences, Tropinina, 49, 603950 Nizhny Novgorod, Russia
[2] Institute of Experimental Oncology and Biomedical Technologies, Privolzhsky Research Medical University, Minin and Pozharsky Sq. 10/1, 603005 Nizhny Novgorod, Russia
[3] Institute of Biology and Biomedicine, Lobachevsky State University of Nizhny Novgorod, 23 Gagarin Avenue, 603950 Nizhny Novgorod, Russia
[4] Becker & Hickl GmbH, Nunsdorfer Ring 7-9, 12277 Berlin, Germany
* Correspondence: komarova.anastasii@gmail.com (A.D.K)
† These authors contributed equally to this work.

Instruments
^{1}H NMR spectra were recorded using Bruker Avance Neo 300 (300 MHz) spectrometer. Chemical shifts are reported relative to the signal of residual protons of the deuterated solvent.
IR spectra were registered using a FTIR FSM 1201 spectrometer. The compounds samples were prepared as thin films on KBr pellets.
Molecular weight distributions were determined using gel permeation chromatography (GPC) using a Knauer chromatograph with Smartline RID 2300 differential refractometer as the detector, with a set of two Phenomenex columns (Phenogel sorbent with pores diameter from 104 to 105 Å, eluent – THF, 2 mL/min, 40°C). The columns were calibrated using 13 polystyrene standards.
The C, H, N elemental analyses were performed at the Microanalytical laboratory of IOMC on a Elementar Vario EL cube elemental analyzer.
The sizes of the polymeric nanoparticles in the aqueous solution were determined from the dynamic light scattering data (a Brookhaven NanoBrook Omni device).

Preparation of organic and iridium-containing monomers

Chart S1. Organic and iridium-containing monomers used for the synthesis of **PIr1-PIr3** probes

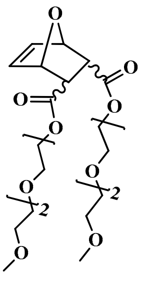

1

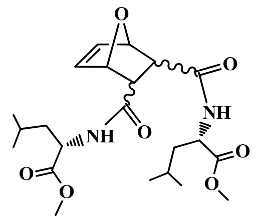

2

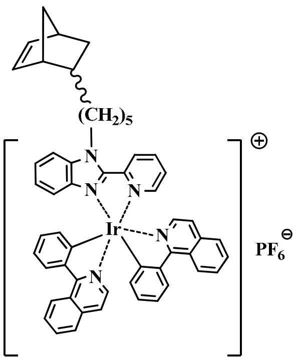

3

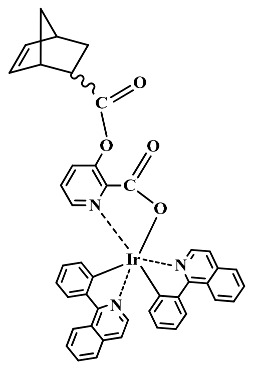

4

Organic monomers **1** [49], **2** [50] were prepared according to the literature procedures. All reactions with air- or moisture-sensitive substances were performed using the standard Schlenk

technique in vacuum or under an argon atmosphere. The reagents and solvents were purchased from commercial sources and used without further purification.

Iridium-containing monomer **3** was synthesized according to the Scheme S1.

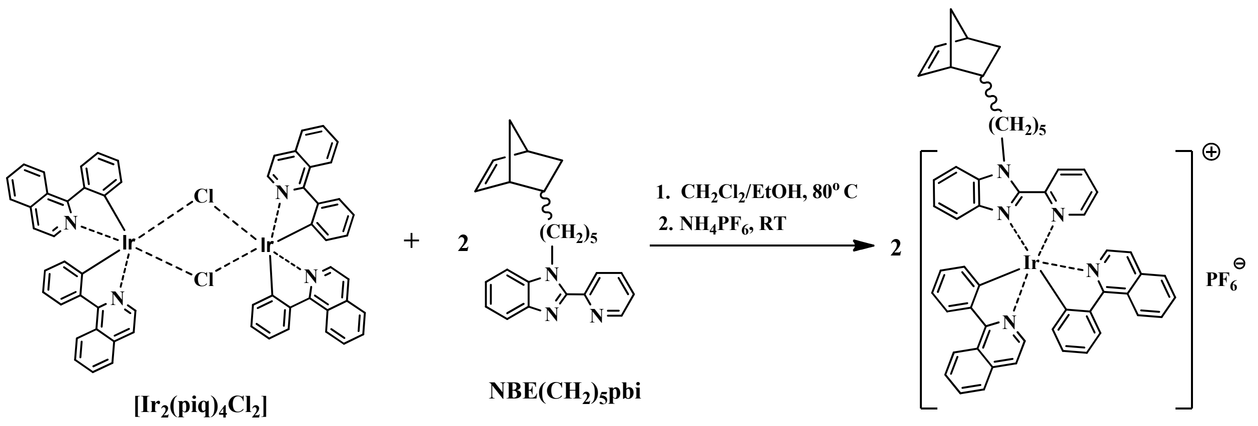

Scheme S1. Synthesis of iridium-containing monomer **3**.

The starting reagents [$Ir_2(piq)_4Cl_2$] [51] and NBE(CH_2)$_5$pbi [52] were synthesized as described in the literature.

Iridium-containing monomer **3**. To a suspension of [$Ir_2(piq)_4Cl_2$] (0.107 g, 0.084 mmol) in a mixture of methanol (4 mL) and dichloromethane (8 mL) NBE(CH_2)$_5$pbi (0.055 g, 0.154 mmol) in dichloromethane (2 mL) was added and the reaction solution was reflux for 8 h. After cooling to room temperature NH_4PF_6 (0.134 g, 0.822 mmol) was added and the reaction mixture was stirred for 2 h. The precipitate was separated by centrifugation, the solution was evaporated and the solid crude product was purified using silica gel column chromatography (CH_2Cl_2/ethyl acetate 10:1 mixture). The iridium-containing monomer **3** was isolated as air stable orange-red solid well soluble in THF, CH_2Cl_2, $CHCl_3$ and insoluble in hexane and H_2O. Yield 0.065 g, 70%. IR, ν, cm^{-1}: 3043 w, 2958 v.s, 2925 v.s, 2852 v.s, 1725 m, 1597 s, 1459 m, 1435 m, 1381 m, 1262 m, 1121 m, 1042 m, 910 w, 841 v.s, 740 m, 557 m. ^1H NMR (300 MHz, $CDCl_3$, δ, ppm): 9.00-8.93 (m, 1H), 8.92-8.85 (m, 1H), 8.57 (d, J = 8.4 Hz, 1H), 8.33-8.21 (m, 3H), 7.90-7.67 (m, 7H), 7.59 (d, J = 6.4 Hz, 1H), 7.52 (d, J = 8.1 Hz, 1H), 7.42-7.28 (m, 5H), 7.21-7.08 (m, 2H), 6.96 (t, J = 7.9 Hz, 1H), 6.89 (t, J = 6.4 Hz, 2H), 6.43 (d, J = 7.7 Hz, 1H), 6.28 (d, J = 7.6 Hz, 1H), 6.10-6.03 (m, 0.9H), 6.02-5.93 (m, 1.2H), 5.87-5.78 (m, 0.9H), 5.06-4.74 (m, 2H), 2.76-2.62 (m, 2H), 2.05 (s, 2H), 1.96-1.44 (m, 5H), 1.22-0.90 (m, 5H), 0.46-0.36 (m, 1H). Anal. Found: C, 58.68; H, 4.25; N, 6.32. Calcd. for $C_{54}H_{47}F_6IrN_5P$: C, 58.79; H, 4.30; N, 6.35.

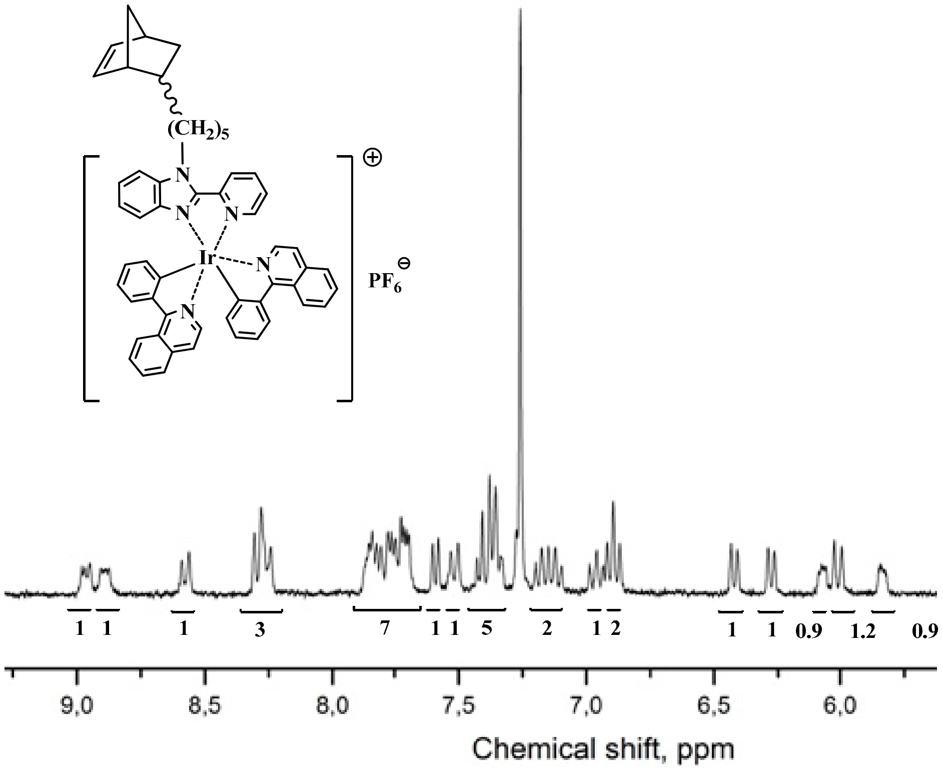

Figure S1. ^{1}H NMR spectra of monomer **3** in $CDCl_3$ (region of aromatic and olefinic protons).

Iridium-containing monomer **4** was synthesized according to the Scheme S2.

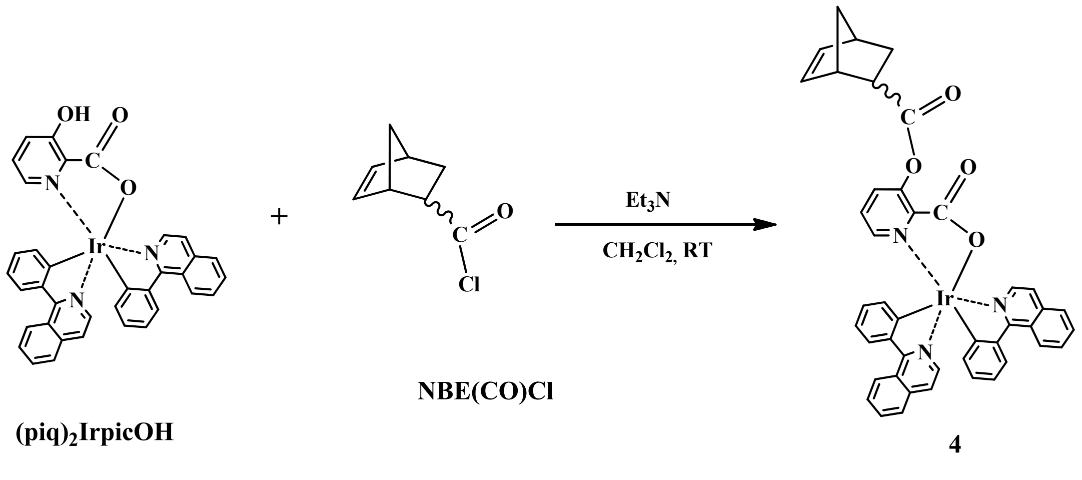

Scheme S2. Synthesis of iridium-containing monomer **4**.

The starting reagents $(piq)_2IrpicOH$ [53] and NBE(CO)Cl [54] were synthesized as described in the literature.

Iridium-containing monomer **4**. A solution of NBE(CO)Cl (0.122 g, 0.780 mmol) in CH_2Cl_2 (5 mL) was added to a solution of $(piq)_2IrpicOH$ (0.126 g, 0.171 mmol) and triethylamine (0.187 g, 1.85 mmol) in CH_2Cl_2 (10 mL) and the mixture was stirred at room temperature for 2 h. Water (20 mL) was added to the reaction solution, organic layer was separated and was dried with magnesium sulfate. After removal of the solvent the solid crude product was purified using silica gel column chromatography (CH_2Cl_2/ethyl acetate 10:1 mixture). The iridium-containing monomer **4** was isolated as air stable dark-red solid well soluble in THF, CH_2Cl_2, $CHCl_3$ and insoluble in hexane and H_2O. Yield 0.111 g, 76%. IR, ν, cm^{-1}: 3045 w, 2962 v.s, 2925 v.s, 2873 v.s, 1655 m, 1578 m, 1441 s, 1383 s, 1331 s, 1267 m, 1150 m, 1105 m, 1045 m, 1026 m,

731 s, 677 s, 517 w. ^{1}H NMR (300 MHz, $CDCl_3$, δ, ppm): 9.00-8.88 (m, 2H), 8.73 (d, J = 6.4 Hz, 1H), 8.25 (d, J = 8.0 Hz, 1H), 8.15 (d, J = 8.0 Hz, 1H), 7.95-7.81 (m, 2H), 7.76-7.66 (m, 4H), 7.54-7.47 (m, 3H), 7.45 (d, J = 6.5 Hz, 1H), 7.33-7.24 (m, 2H), 7.00 (t, J = 7.8 Hz, 1H), 6.90 (t, J = 7.8 Hz, 1H), 6.77 (t, J = 7.4 Hz, 1H), 6.67 (t, J = 7.4 Hz, 1H), 6.48 (d, J = 7.8 Hz, 1H), 6.20-6.05 (m, 3H), 3.47 (s, 0.5H), 3.42 (s, 0.5H), 2.98-2.87 (m, 1H), 2.72-2.61 (m, 1H), 2.27-2.13 (m, 1H), 1.66-1.36 (m, 3H). Anal. Found: C, 61.55; H, 3.93; N, 4.78. Calcd. for $C_{44}H_{32}IrN_3O_4$: C, 61.52; H, 3.76; N, 4.89.

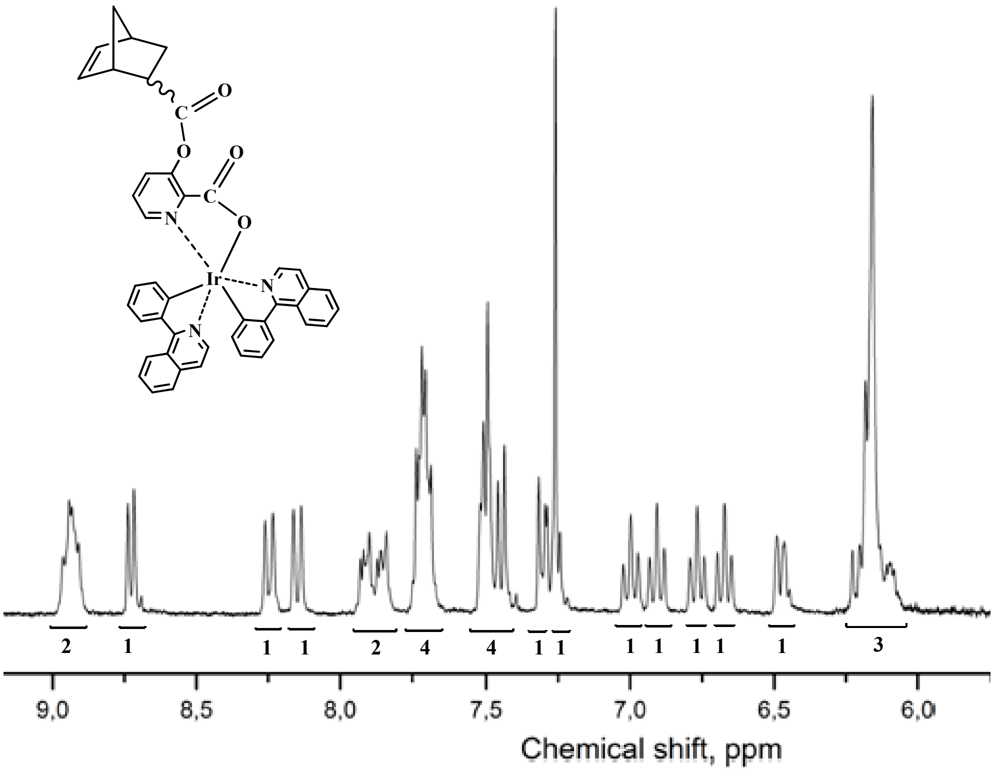

Figure S2. ^{1}H NMR spectra of monomer **4** in $CDCl_3$ (region of aromatic and olefinic protons).

Table S1. Photophysical characteristics of monomers **3** and **4** in CH_2Cl_2 solution

Monomer	λ_{max}^{abs}/nm (log ε) in CH_2Cl_2	λ_{max}^{em}/nm (in CH_2Cl_2)	Quantum yield, % (in CH_2Cl_2) a)	b)	Chromaticity coordinates in the CIE diagram (*x*; *y*)
**3**	291 (5.33), 341 (5.18), 381 (4.96), 448 (4.78), 484 (4.64)	588, 629 sh	18.69	3.68	0.57; 0.41
**4**	297 (5.06), 348 (4.83), 404 (4.52), 466 (4.38), 510 (4.18)	605	11.49	2.47	0.62; 0.38

a) Degassed solution.
b) Aerated solution.

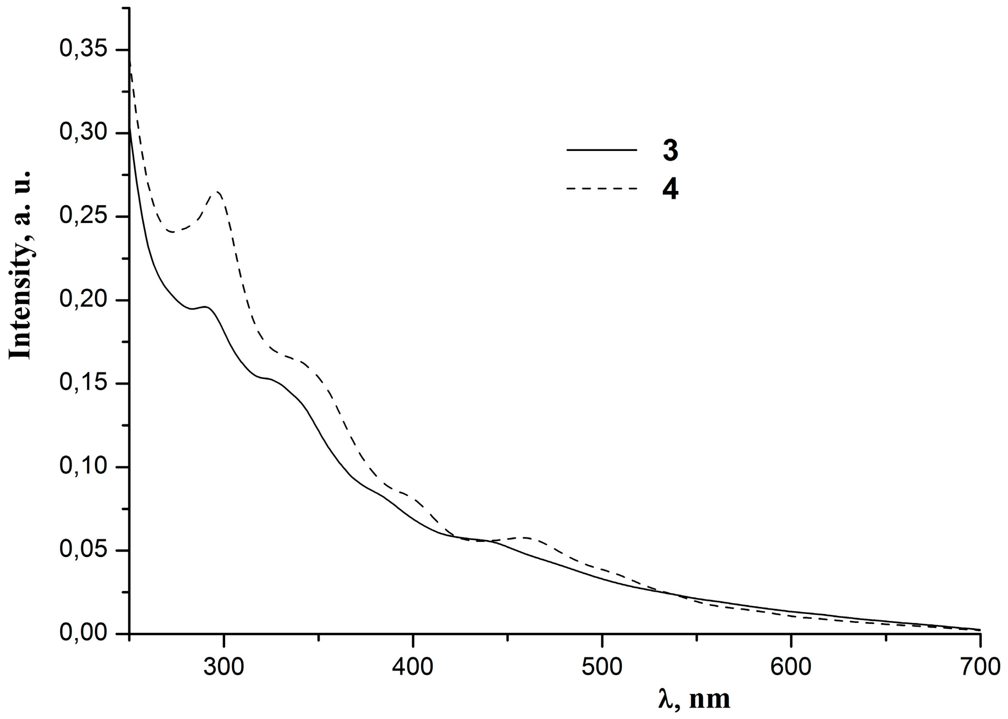

Figure S3. Absorption spectra of monomers **3** and **4** in CH_2Cl_2 solution.

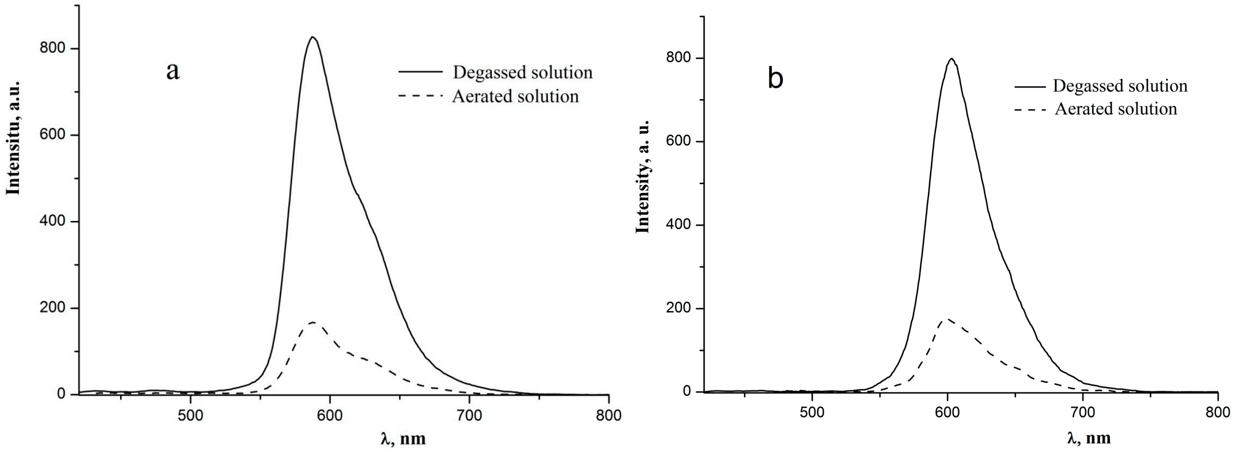

Figure S4. Photoluminescence (PL) spectra of monomers **3** (a) and **4** (b) in CH_2Cl_2 solution at room temperature, λ_{ex} = 360 nm.

Preparation of polymeric probes PIr1-PIr3

Polymeric probes **PIr1** and **PIr2** were synthesized according to the Scheme S3.

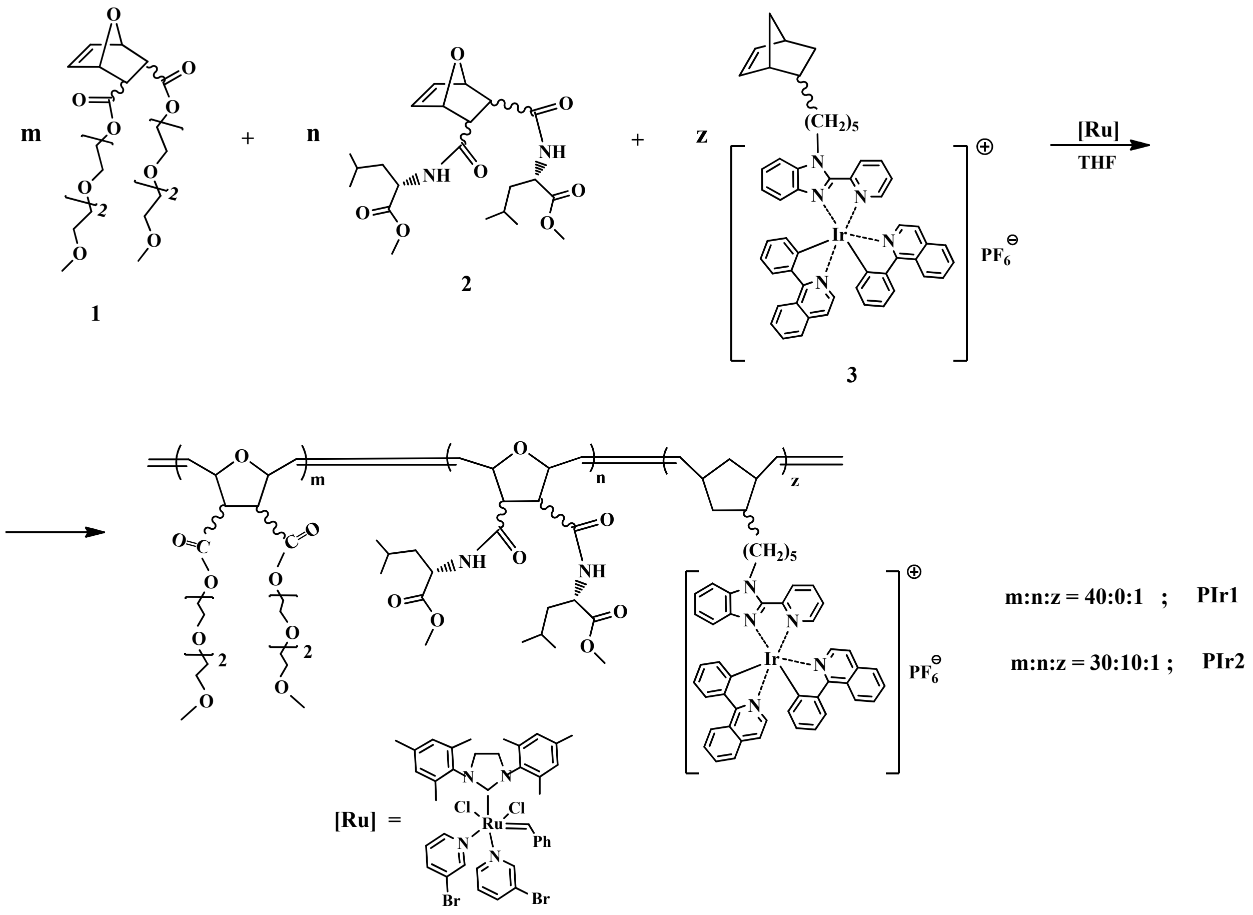

Scheme S3. Synthesis of **PIr1** and **PIr2** probes.

Third generation Grubbs catalyst (H_2IMes)(3-Brpy)$_2Cl_2$Ru=CHPh, used in the synthesis of iridium-containing polymeric probes was prepared as described in the literatures [55, 56].

Polymeric probe **PIr1**. Organic monomer **1** (0.1139 g, 0.239 mmol) in 1 mL of THF was placed into evacuated ampoule. Iridium-containing monomer **3** (0.0065 g, 0.006 mmol) in 0.5 mL of THF was added to the solution of monomer **1** and finally 0.0026 g (0.003 mmol) of third-generation Grubbs catalyst in 0.5 mL of THF was added to the mixture of monomers **1** and **3** at room temperature. The solution of monomers and catalyst was magnetically stirred at 40°C. The reaction was monitored by thin layer chromatography and it was found that polymerization completed in 10 h. The reaction mixture was cooled to room temperature and after addition of few drops of ethyl vinyl ether was stirred for 30 min. The reaction solution was added drop-wise into stirring hexane (40 mL) and precipitated polymeric product was isolated by centrifugation and dried in vacuum at room temperature to a constant weight. The yield of polymeric probe **PIr1** (brown gummy substance) was 0.112 g (93%). IR, ν, cm^{-1}: 2948 v.s, 2918 v.s, 2879 v.s (C_{alif}-H); 1743 v.s (C=O); 1455 m, 1352 m, 1285 m, 1198 s, 1109 s, 1036 m (C-O, C-H), 978 m, 847 m, 758 w. ^{1}H NMR (300 MHz, $CDCl_3$, δ, ppm): 9.10-8.80 (m, 2H), 8.60-8.20 (m, 4H), 8.00-7.30 (m, 14H), 7.20-6.80 (m, 5H), 6.50-6.00 (m, 4H), 5.95-5.76 (m, 41H), 5.64-5.46 (m, 40H), 5.16-4.94 (m, 42H), 4.76-4.58 (m, 40H), 4.38-4.06 (m, 160H), 3.70-3.60 (m, 640H), 3.58-3.50 (m, 160H), 3.36 (br. s, 240H), 3.20-3.00 (m, 82H), 1.90-1.80 (m, 7H), 1.30-1.10 (m, 6H). Anal. Found: C, 55.33; H, 7.43; N, 0.31. Calcd for $C_{934}H_{1487}F_6IrO_{440}N_5P$: C, 55.65; H, 7.38; N, 0.35. M_w = 32300, M_n = 22000, M_w/M_n = 1.47.

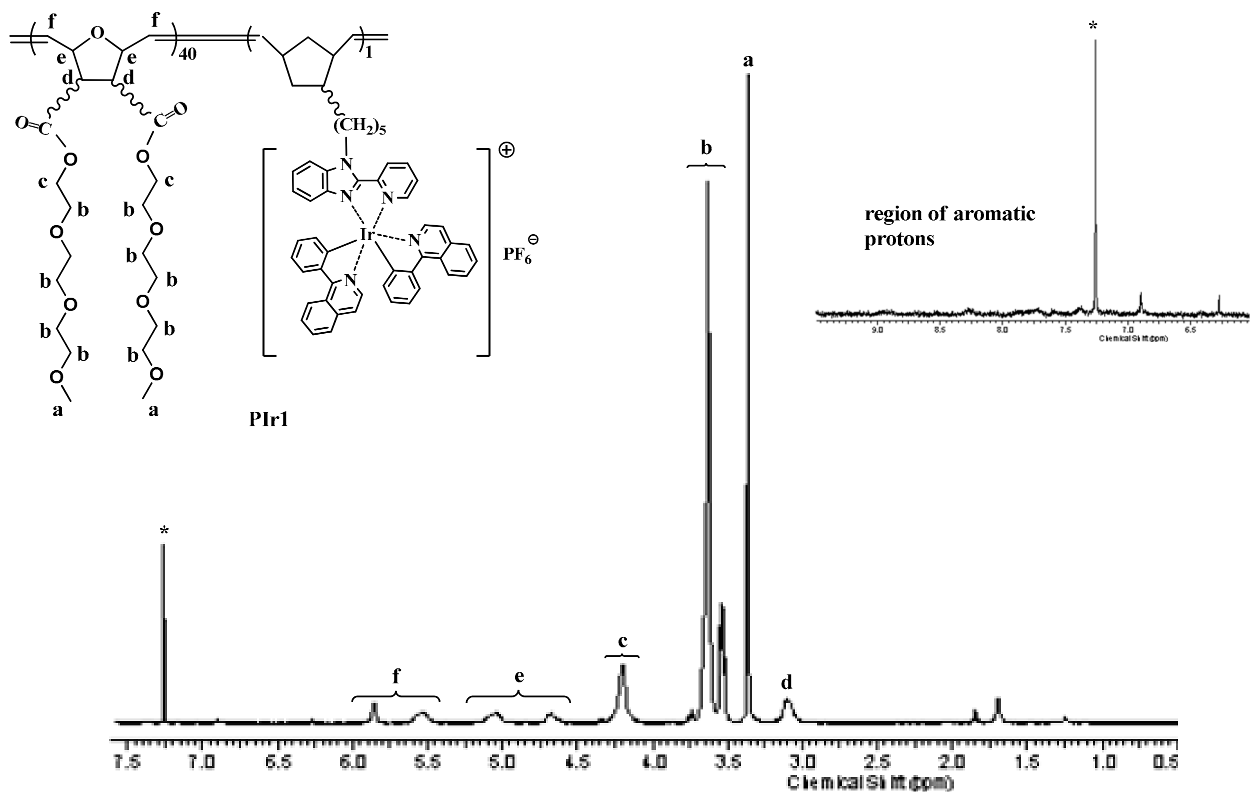

Figure S5. ^{1}H NMR spectra of polymeric probe **PIr1** in $CDCl_3$. (*) The solvent residual signal.

Polymeric probe **PIr2**. Organic monomers **1** (0.1402 g, 0.294 mmol) and **2** (0.0429 g, 0,098 mmol) and iridium-containing monomer **3** (0.0108 g, 0.010 mmol) in 2 mL of THF were placed into evacuated ampoule. Third-generation Grubbs catalyst (0.0036 g, 0.004 mmol) in 0.5 mL of THF was added to the mixture of monomers **1**, **2** and **3** at room temperature. The reaction solution was magnetically stirred at 40°C. The polymerization was monitored by thin layer chromatography and was found to complete in 10 h. The reaction mixture was cooled to room temperature and after addition of few drops of ethyl vinyl ether was stirred for 30 min. The reaction solution was added drop-wise into stirring hexane (40 mL) and precipitated polymeric product was isolated by centrifugation and dried in vacuum at room temperature to a constant weight. The yield of polymeric probe **PIr2** (brown gummy substance) was 0.186 g (96%). IR, ν, cm^{-1}: 3327 m (N-H); 2956 v.s, 924 v.s, 2871 v.s (C_{alif}-H); 1742 v.s, (C=O); 1540 m (N-H); 1455 m, 1354 m, 1285 m, 1254 m, 1202 s, 1112 s, 1032 m (C-O, C-H); 980 m, 849 m, 752 w. ^{1}H NMR (300 MHz, $CDCl_3$, δ, ppm): 9.10-8.70 (m, 2H), 8.60-8.20 (m, 4H), 8.00-7.30 (m, 14H), 7.20-6.80 (m, 5H), 6.70-6.20 (m, 4H), 6.00-5.70 (m, 41H), 5.65-5.35 (m, 40H), 5.30-4.85 (m, 42H), 4.80-4.45 (m, 40H), 4.35-4.06 (m, 140H), 3.75-3.56 (m, 540H), 3.56-3.46 (m, 120H), 3.36 (br. s, 200H), 3.22-2.98 (m, 82H), 1.90-1.46 (m, 67H), 1.28-1.22 (m, 6H), 0.98-0.82 (m, 120H). Anal. Found: C, 56.42; H, 7.50; N, 1.79. Calcd for $C_{934}H_{1467}F_6IrO_{400}N_{25}P$: C, 56.71; H, 7.42; N, 1.77. M_w = 22500, M_n = 17200, M_w/M_n = 1.31.

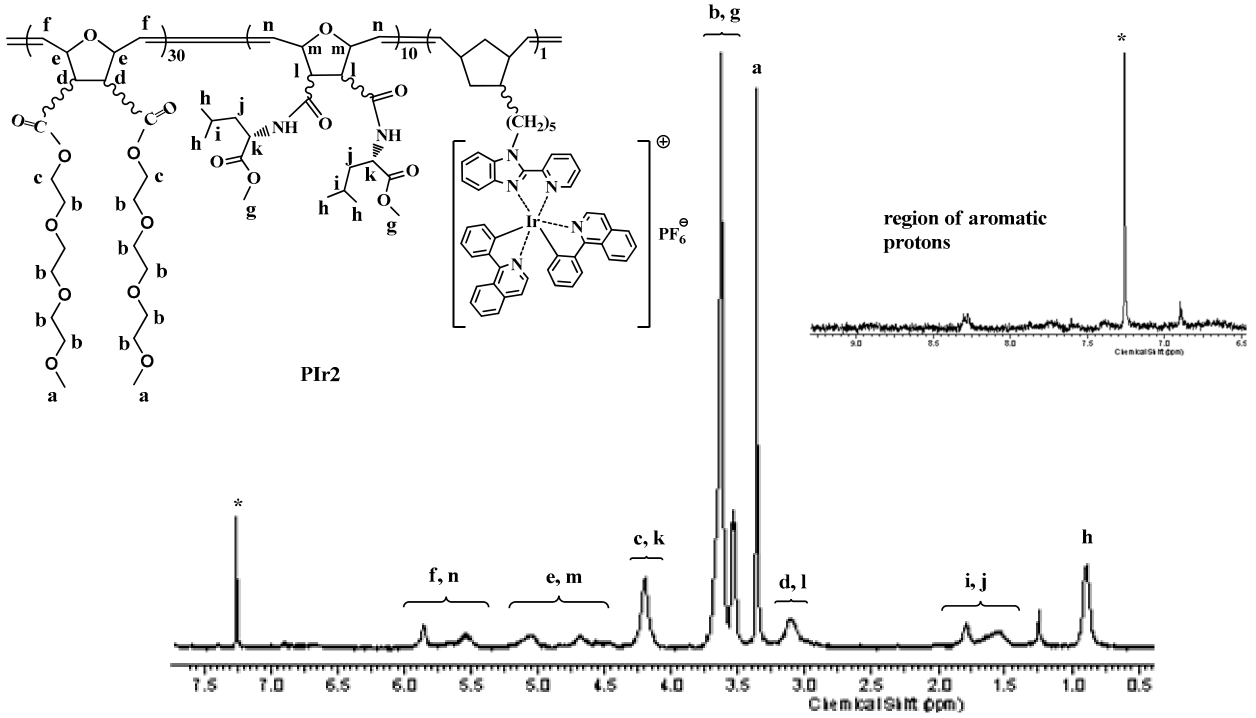

Figure S6. ^{1}H NMR spectra of polymeric probe **PIr2** in $CDCl_3$. (*) The solvent residual signal.

Polymeric probes **PIr3** was synthesized according to the Scheme S4.

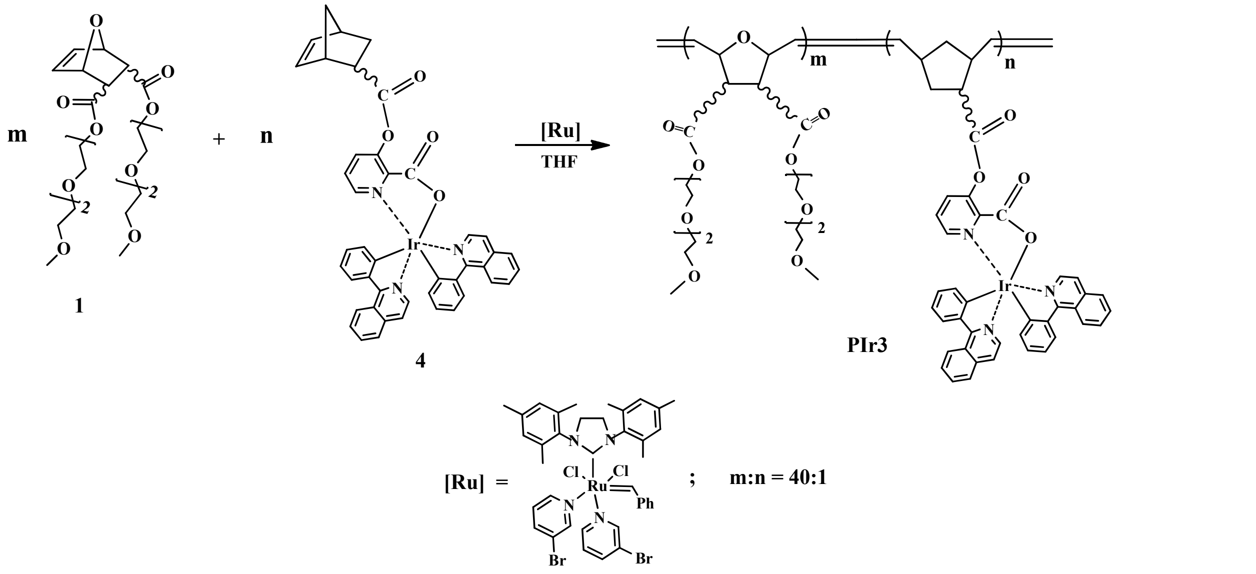

Scheme S4. Synthesis of **PIr3** probe.

Polymeric probe **PIr3**. Organic monomer **1** (0.1213 g, 0.255 mmol) in 1 mL of THF was placed into evacuated ampoule. Iridium-containing monomer **4** (0.0055 g, 0.0064 mmol) in 0.5 mL of THF was added to the solution of monomer **1** and finally 0.0025 g (0.0028 mmol) of third-generation Grubbs catalyst in 0.5 mL of THF was added to the mixture of monomers **1** and **4** at room temperature. The solution of monomers and catalyst was magnetically stirred at 40°C. The reaction was monitored by thin layer chromatography and it was found that polymerization completed in 10 h. The reaction mixture was cooled to room temperature and after addition of few drops of ethyl vinyl ether was stirred for 30 min. The reaction solution was added drop-wise into stirring hexane (40 mL) and precipitated polymeric product was isolated by centrifugation and dried in vacuum at room temperature to a constant weight. The yield of polymeric probe **PIr3** (orange-red gummy substance) was 0.119 g (94%). IR, ν, cm^{-1}: 2947 v.s, 2916 v.s, 2879 v.s (C_{alif}-H); 1742 v.s (C=O); 1455 m, 1350 m, 1285 m, 1198 s, 1109 s, 1038 m (C-O), 978 m, 852

m, 760 w. ^{1}H NMR (300 MHz, $CDCl_3$, δ, ppm): 9.00-8.60 (m, 3H), 8.30-7.85 (m, 4H), 7.80-7.30 (m, 10H), 7.00-6.40 (m, 5H), 6.00-5.75 (m, 43H), 5.70-5.35 (m, 40H), 5.20-4.85 (m, 40H), 4.80-4.50 (m, 40H), 4.40-4.05 (m, 160H), 3.75-3.58 (m, 640H), 3.56-3.48 (m, 161H), 3.36 (br. s, 240H), 3.18-3.00 (m, 83H), 1.90-1.80 m (3H). Anal. Found: C, 55.41; H, 7.43; N, 0.24. Calcd for $C_{924}H_{1472}IrO_{444}N_3$: C, 55.72; H, 7.40; N, 0.21. M_w = 62600, M_n = 39300, M_w/M_n = 1.59.

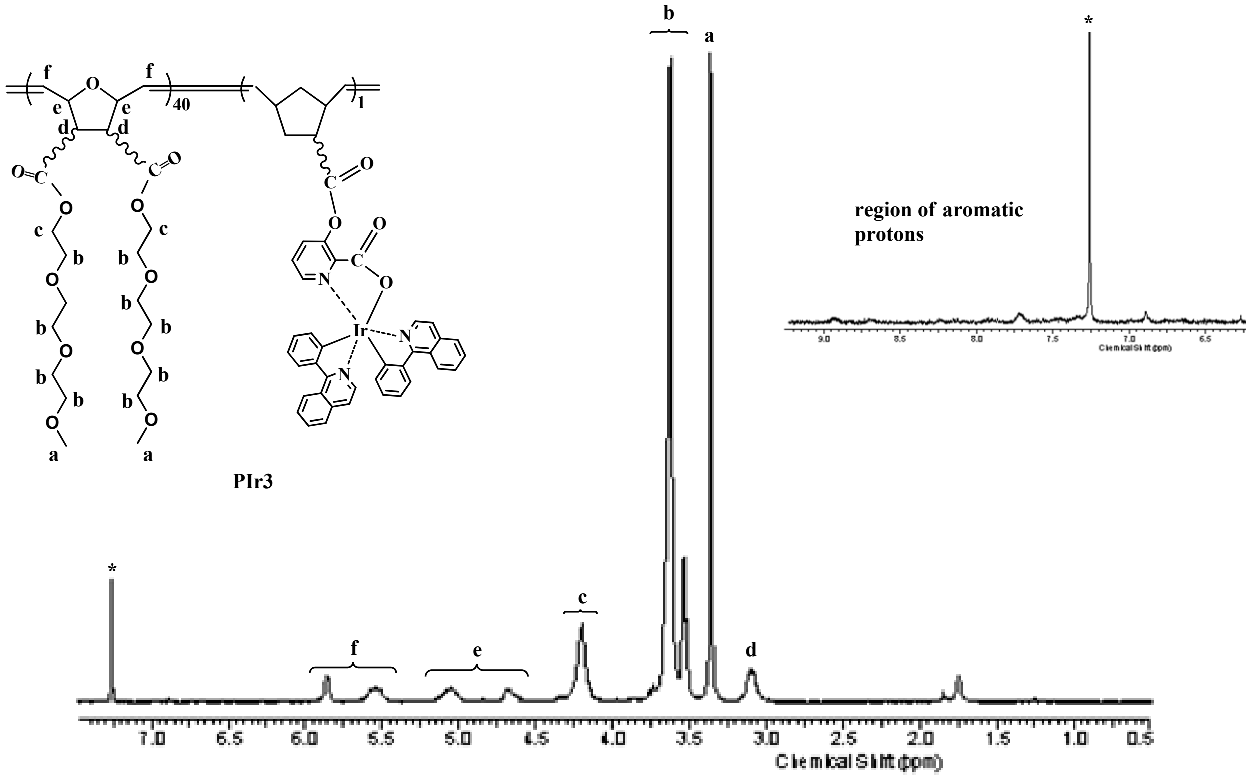

Figure S7. ^{1}H NMR spectra of polymeric probe **PIr3** in $CDCl_3$. (*) The solvent residual signal.

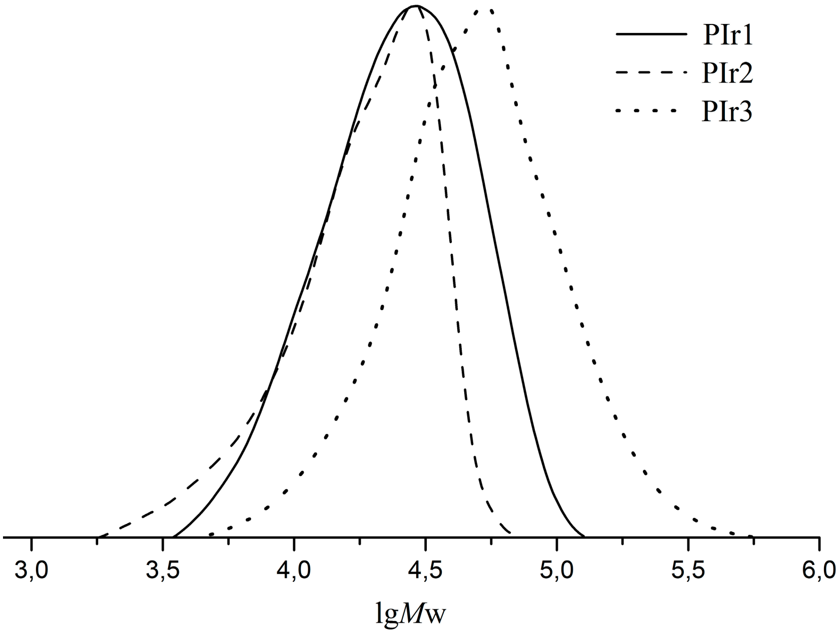

Figure S8. Normalized GPC curves of polymeric probes **PIr1-PIr3**.

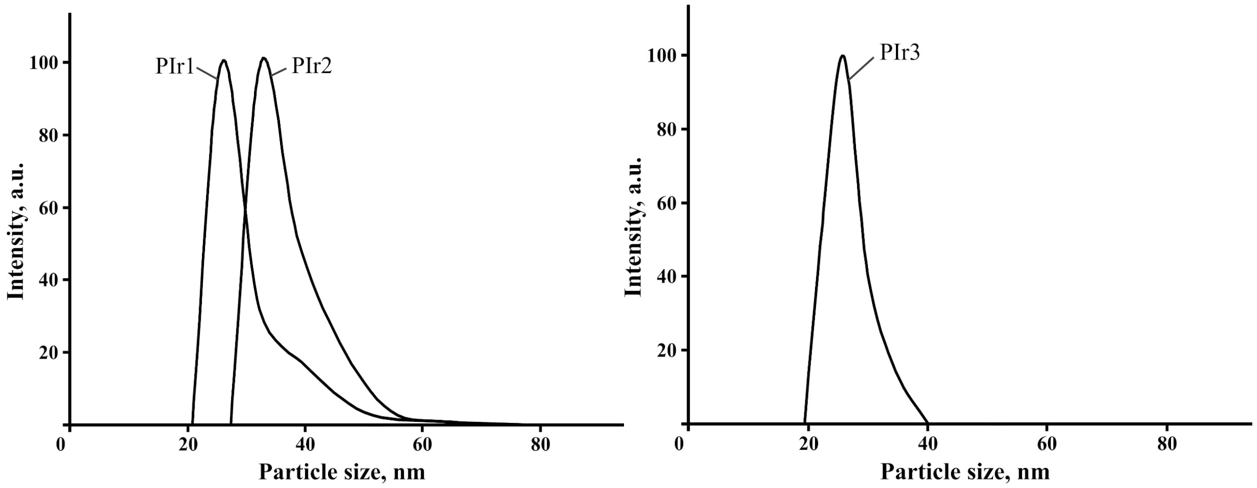

Figure S9. Particle size distribution of polymeric probes **PIr1-PIr3** in aqueous solutions.

Simultaneous PLIM and FLIM *in vivo*

Upon intravenous injection at the dose of 40 mg/kg the polymer probe PIr3 accumulated in the skin, which was detected by PLIM. The developed PLIM/FLIM protocol was used to assess in vivo metabolism and oxygenation in normal epithelial cells of the skin in the mouse ear (Fig. S10). Skin cells were characterized by a decreased phosphorescence lifetime of the probe PIr3 1.5 ± 0.09 µs (n=50 cells) and a decreased contribution of free NAD(P)H a_1 67.4 ± 1.7 %, compared to tumor cells, which suggest a correlative shift towards oxidative phosphorylation in the presence of adequate oxygen content. No correlation was found between τ_m PIr3 and a_1 NAD(P)H in skin cells (r=0.03). Unlike tumor cells, the population of skin epithelium cells was more uniform in terms of oxygen distribution and metabolism.

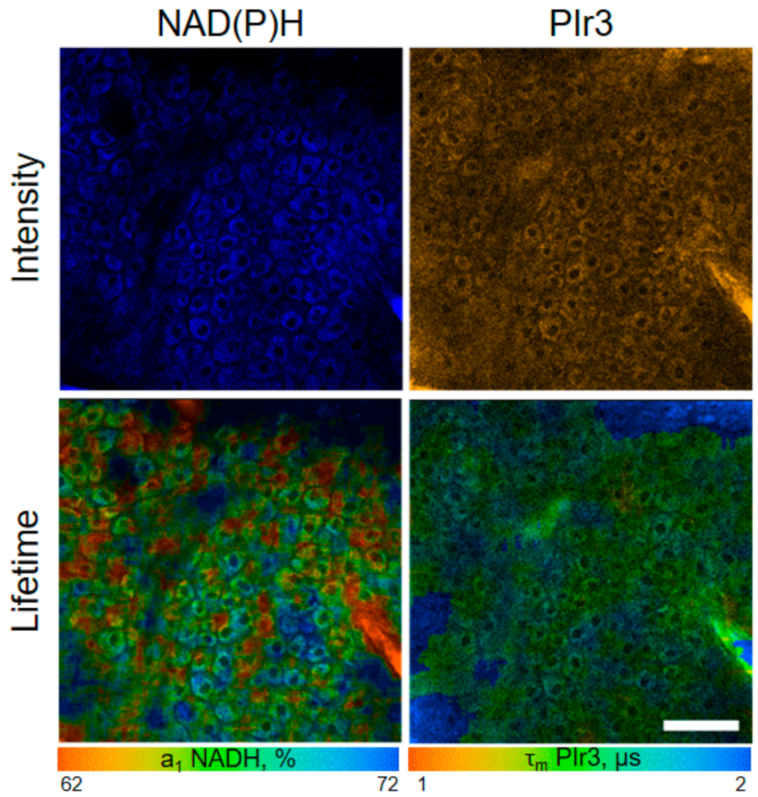

Figure S10. In vivo assessment of oxygen and metabolic statuses in the mouse skin using simultaneous PLIM/FLIM technique. Images were obtained at 6 hours post-i.v. injection of the PIr3 probe. Scale bar: 50 µm.

Table S2. Fluorescence lifetime parameters of metabolic cofactor NAD(P)H in the CT26 mouse tumors. Median (Q1; Q3).

Tumor volume, mm^3	PIr3, μs	a_1 NAD(P)H, %	τ_m NAD(P)H, μs	τ_1 NAD(P)H, μs	τ_2 NAD(P)H, μs
< 7	1.77 (1.53; 1.79)	81.67 (80.39; 82.81)	0.76 (0.74; 0.79)	0.39 (0.38; 0.41)	2.45 (2.23; 2.51)
13-20	1.88 (1.82; 1.95)	84.01 (83.52; 85.08), $p=0.005$ with tumors <7 mm^3 $p=5.1x10^{-5}$ with tumors > 40 mm^3	0.71 (0.69; 0.74), $p=1.8x10^{-5}$ with tumors <7 mm^3 $p=0.027$ with tumors > 40 mm^3	0.41 (0.39; 0.41)	2.35 (2.30; 0.24)
> 40	1.71 (1.19; 1.74)	80.24 (78.98; 83.14)	0.72 (0.66; 0.76)	0.37 (0.35; 0.39)	2.30 (2.20; 2.43)
